# Impact of pre-sarcopenia in sorafenib treatment for advanced hepatocellular carcinoma

**DOI:** 10.1371/journal.pone.0198812

**Published:** 2018-06-18

**Authors:** Hitomi Takada, Masayuki Kurosaki, Hiroyuki Nakanishi, Yuka Takahashi, Jun Itakura, Kaoru Tsuchiya, Yutaka Yasui, Nobuharu Tamaki, Kenta Takaura, Yasuyuki Komiyama, Mayu Higuchi, Youhei Kubota, Wann Wang, Mao Okada, Nobuyuki Enomoto, Namiki Izumi

**Affiliations:** 1 Department of Gastroenterology and Hepatology, Musashino Red Cross Hospital, Tokyo, Japan; 2 First Department of Internal Medicine, Faculty of Medicine, University of Yamanashi, Yamanashi, Japan; Taipei Veterans General Hospital, TAIWAN

## Abstract

**Background:**

The present study aimed to investigate the impact of pre-sarcopenia on the prognosis of patients with advanced hepatocellular carcinoma (HCC) treated with sorafenib.

**Methods:**

We enrolled 214 patients (71 ± 10 years old; 166 men and 48 women; 90% Child–Pugh grade A and 10% Child–Pugh grade B) treated with sorafenib in our hospital from July 2009 to August 2016. The muscle volume was measured from CT images just before sorafenib administration using software (SliceOmatic). Skeletal muscle mass index was calculated, and the presence of pre-sarcopenia was judged according to the standard (42 cm^2^/m^2^ for men and 38 cm^2^/m^2^ for women) proposed by the Japan Society of Hepatology.

**Results:**

Pre-sarcopenia was found in 123 patients (57%). The overall survival (OS) in patients with pre-sarcopenia tended to be worse than in patients without pre-sarcopenia (median 252 vs. 284 days, respectively; *p* = 0.16). Multivariate Cox hazard analysis revealed a baseline serum albumin level of ≤3.5 g/dl [hazard ratio (HR) 1.9; *p* = 0.0006], a baseline alpha-fetoprotein(AFP) level of ≥100 ng/ml (HR 2.1; *p* = 0.002), presence of lesions in bilateral hepatic lobes (HR 1.7; *p* = 0.03), and presence of major portal vein invasion (HR 1.8; *p* = 0.01) to be independent prognostic factors. In the 68 patients who had three or more negative prognostic factors, the presence of pre-sarcopenia did not correlate with prognosis. Of the 146 patients who had two or less prognostic factors, OS was significantly worse in 84 patients (58%) with pre-sarcopenia than in 62 patients without pre-sarcopenia (median 417 vs. 562 days, respectively; *p* = 0.047), and Cox hazard analysis revealed pre-sarcopenia to be an important prognostic factor (HR 1.6; *p* = 0.047).

**Conclusion:**

In sorafenib treatment for advanced HCC, pre-sarcopenia is a significant prognostic factor in patients with two or less negative prognostic factors, and could be the target of intervention to improve prognosis.

## Introduction

Sarcopenia is defined as loss of both muscle volume and function (loss of muscle strength or physical function), and loss of muscle volume alone has specifically been defined as pre-sarcopenia. Sarcopenia is classified into primary sarcopenia (associated with aging) and secondary sarcopenia (associated with chronic disease or malignant tumors) [1]. Among the various causes of secondary sarcopenia, chronic liver disease and hepatocellular carcinoma (HCC) are regarded as high-risk diseases. In fact, it has been reported that sarcopenia is present even in non-elderly patients with chronic liver disease. The prevalence of sarcopenia is reported to be 15% in patients with chronic hepatitis [2], up to 40% in patients with compensated cirrhosis [3], and 11%–45% in patients with HCC [4, 5]. Notably, sarcopenia has attracted attention as a factor predicting poor prognosis, and there is increasing evidence supporting the importance of sarcopenia and pre-sarcopenia in patients with chronic liver disease and HCC [6–9]. The impact of sarcopenia on the prognosis of patients with HCC has been reported in several studies. The presence of sarcopenia correlated with poor prognosis in patients after surgical resection of HCC [10, 11], liver transplantation [12], or intra-arterial therapy [13].

Treatment of advanced HCC that is not amenable to resection, liver transplantation, or locoregional therapy, such as radiofrequency ablation or transcatheter arterial chemoembolization (TACE), remains a challenge in clinical practice. Sorafenib is a drug that has been confirmed to be effective in improving survival of patients with advanced stage HCC [14] and is now recommended as the first-line treatment option for advanced HCC that is refractory to TACE or does not meet the criteria for resection, transplantation, or locoregional therapy. Accordingly, there is a wide range of HCC conditions among patients treated with sorafenib, such as presence or absence of metastasis, major vascular invasion, tumor volume, and serum biomarkers. However, information is limited on the prevalence and significance of sarcopenia in patients with advanced HCC treated with sorafenib [15–17], and no previous study has analyzed the impact of sarcopenia in subgroups of patients stratified by HCC conditions. In the present study, we investigated the impact of pre-sarcopenia as a prognostic factor in patients with advanced HCC treated with sorafenib after stratification by standard prognostic factors.

## Methods

### Patients

This is a retrospective cohort study that enrolled 214 consecutive patients treated with sorafenib in our hospital from July 2009 to August 2016. This study was approved by the institutional ethics committee (Ethics committee for clinical studies of Musashino Red Cross Hospital: Rinshoukenkyu-Rrinrishinsa-Iinkai (in Japanese), approved number 185) in accordance with the Declaration of Helsinki. The diagnosis of HCC was based on histology or radiological findings, such as arterial enhancement, followed by a washout in the portal venous phase, as viewed on dynamic computed tomography (CT) imaging or magnetic resonance imaging (MRI), in accordance with the criteria of practice guidelines [18]. The definition of major portal vein invasion is invading the main trunk/the first-order branch of the portal vein (Vp4/3). Inclusion criteria for sorafenib treatment were having metastatic or locally advanced HCC that was unresectable or refractory to TACE, Barcelona Clinic Liver Cancer stage B or C [19], and Eastern Cooperative Oncology Group performance status 0 to 1 [20]. Patients with active infections, those with poorly controlled thromboembolic disease, and those with interstitial pneumonia were excluded.

### Sorafenib treatment

All study participants provided informed consent for treatment and for the participation in the clinical study, and then treatment was commenced. The initial dose of sorafenib was 800 mg/day in general but was reduced to 400 mg/day for patients with advanced age (≥80 years), Child–Pugh grade B, low body weight (<50 kg), or having pleural effusion/ascites or gastrointestinal varices with a risk of bleeding[21–25]. The initial dose of sorafenib was reduced by the judgement of physicians in order to avoid early discontinuation due to severe adverse events. We have previously reported that prognosis was comparable between patients treated with standard 800mg of sorafernib and those with reduced dose[24]. There are some reports that the duration of sorafenib administration appears to influence prognosis more than initial dose[22–25]. These were the rationales for the dose reduction. CT imaging was performed before and one month after starting sorafenib and every three months thereafter. Radiological responses to therapy were evaluated according to modified Response Evaluation Criteria in Solid Tumors (modified RECIST)[26, 27]. Serial measurements of blood samples were performed before and monthly after sorafenib treatment. Sorafenib was discontinued and palliative treatments or best supportive care was provided in cases of radiological progressive disease or intolerable adverse events. Patients with combination therapy were not included, and all the patients received sorafenib monotherapy. In this study, patients who had experience of other systemic treatments were excluded.

### Muscle volume measurement

The muscle volume was measured from CT images just before sorafenib administration using software (SliceOmatic) [28]. Skeletal muscle mass index, which is the sum of muscle area at the level of third lumbar vertebra (L3) divided by the square of the height, was calculated and the presence of pre-sarcopenia was judged according to the standard (42 cm^2^/m^2^ for men and 38 cm^2^/m^2^ for women) proposed by the Japan Society of Hepatology [29]. Both diagnosis and evaluation were performed blindly by two trained hepatologists with knowledge about radiological anatomy and experience in skeletal muscle area measurements.

### Survival analysis

The endpoint was set at overall survival (OS). OS was calculated from the initial date of sorafenib treatment until death due to any cause or until the last follow-up. Baseline factors associated with OS were analyzed. The factors analyzed for prognostic significance included age, gender, serum data (albumin, alpha-fetoprotein (AFP), Lens culinaris agglutinin-reactive fraction of AFP (AFP L3 fraction)[30, 31], and prothrombin induced by vitamin K absence II (PIVKA-II) levels) before sorafenib administration, HCC conditions (maximum diameter of intrahepatic HCC, tumor number, tumor location, major vessel invasion, and distant metastasis), and presence of pre-sarcopenia. Furthermore, we also analyzed the correlation between the presence or absence of pre-sarcopenia and OS after stratification by standard prognostic factors.

### Statistical analysis

Categorical variables were analyzed using Fisher’s exact test, and continuous variables were compared using the unpaired Student’s t-test. *P* value <0.05 was considered statistically significant. Data were expressed as mean±standard deviation. OS was evaluated by Kaplan–Meier curves and differences between groups were assessed using the log-rank test. A Cox proportional hazards model was used to determine the factors associated with OS. All statistical analyses were performed using the statistical analysis software R (http://www.r-project.org) [32].

## Results

### Patient background

Patient backgrounds are shown in Table 1. Values are mean±standard deviation. Baseline liver function was Child–Pugh grade A in 192 patients (90%) and grade B in 22 patients (10%). Serum albumin level was ≤3.5 g/dl in 95 patients (44%). Overall, 38 patients (19%) had received sorafenib as initial therapy for HCC, 76 patients (35%) showed evidence of extrahepatic metastasis, 47 patients (22%) had major portal vein invasion, 152 patients (70%) had lesions in bilateral hepatic lobes, and the maximum size of tumor in the liver was ≥60 mm in 71 patients (35%). Regarding serum biomarkers, elevated AFP levels (≥100 ng/ml), AFP L3 fraction (≥10%), and PIVKA-II levels (≥100 mAU/ml) were found in 120 (56%), 125 (58%), and 136 (65%) patients, respectively.

**Table 1:** Backgrounds of patients treated with sorafenib.

Age (years)	71 ± 10
Males	166 (77%)
Albumin (g/dl)	3.5 ± 0.5
Total bilirubin (mg/dl)	0.81 ± 0.42
Prothrombin time (%)	95 ± 18
Child–Pugh Class A/B	192 (90%) / 22 (10%)
Sorafenib as initial treatment for HCC	38 (19%)
AFP (ng/ml)	5648 ± 14962
AFP L3 fraction (%)	30 ± 27
PIVKA-II (mAU/ml)	21653 ± 158674
Maximum size of tumor in the liver (mm)	50 ± 42
Lesions in bilateral hepatic lobes	152 (70%)
Major portal vein invasion	47 (22%)
Extrahepatic metastasis	76 (35%)
Pre-sarcopenia	123 (57%)

Continuous values are expressed as mean ± standard deviation. AFP, alpha-fetoprotein; AFP L3 fraction, Lens culinaris agglutinin-reactive fraction of AFP; PIVKA-II, prothrombin induced by vitamin K absence II.

### Prevalence of pre-sarcopenia

Pre-sarcopenia was found in 123 patients (57%). Patients with pre-sarcopenia were older (74 ± 8 vs. 68 ± 11 years, respectively; *p* < 0.001) and the prevalence in females was higher (32% vs. 12%, respectively; *p* = 0.001), compared with those without pre-sarcopenia. Other baseline factors including results of blood tests, status of HCC, and serum biomarkers were not different between groups (Table 2).

**Table 2:** Comparison of backgrounds in patients with and without pre-sarcopenia.

	With pre-sarcopenia (n = 123)	Without pre-sarcopenia (n = 91)	*P* value
Age (years)	74 ± 8	68 ± 11	< 0.001
Males	84 (68%)	82 (88%)	0.001
Albumin (g/dl)	3.5 ± 0.49	3.5 ± 0.52	0.48
Total bilirubin (mg/dl)	0.91 ± 0.56	0.96 ± 0.51	0.52
Prothrombin time (%)	97 ± 18	93 ± 16	0.085
Child–Pugh Class A/B	111 (90%)/12 (10%)	80 (88%)/11 (12%)	0.57
Sorafenib as initial treatment for HCC	19 (17%)	18 (20%)	0.24
AFP (ng/ml)	6810 ± 17533	4112 ± 10550	0.19
AFP L3 fraction (%)	30 ± 28	29 ± 26	0.73
PIVKA-II (mAU/ml)	30531 ± 208187	10044 ± 37430	0.36
Maximum size of tumor in the liver (mm)	51 ± 42	48 ± 40	0.62
Lesions in bilateral hepatic lobes	85 (69%)	67 (72%)	0.66
Major portal vein invasion	27 (22%)	20 (22%)	1.0
Extrahepatic metastasis	38 (31%)	38 (41%)	0.15

Continuous values are expressed as mean ± standard deviation. AFP, alpha-fetoprotein; AFP L3 fraction, Lens culinaris agglutinin-reactive fraction of AFP; PIVKA-II, prothrombin induced by vitamin K absence II.

### Overall survival

**Fig 1:**
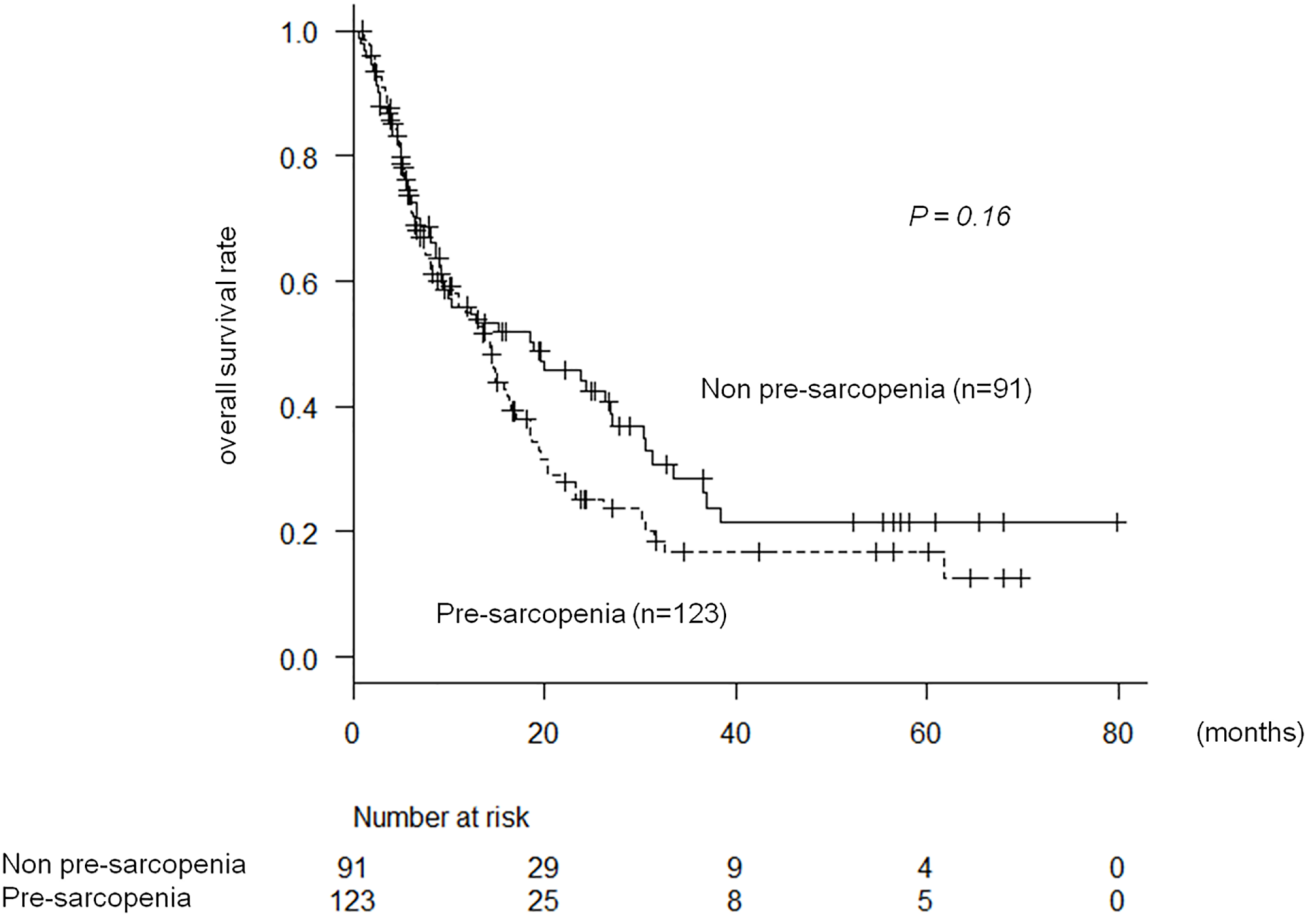
Pre-sarcopenia and overall survival in patients undergoing sorafenib therapy.

The median survival time (MST) after sorafenib administration was 14.4 months (95% confidence interval (CI) 10.1–17.1 months). OS in patients with pre-sarcopenia tended to be worse than in patients without pre-sarcopenia; MST was 13.7(8.8–16) vs. 18.5(9.1–26) months and 6-, 12-, and 18-month survival rates were 72%, 53%, and 37% vs. 72%, 55, and 50%, respectively (p = 0.16) (Fig 1).

### Sorafenib treatment and dose reduction

Median duration of sorafenib administration was 3.3 months (0.17–79 months). There was a correlation between sorafenib treatment duration and survival time. Patients taking sorafenib for more than 3 months (n = 124) had longer survival time than cases of 3 months or less (n = 90) (19(15–23) vs. 6.5(4.9–8.1) months; p < 0.0001). One-hundred fifty six patients experienced reduction of sorafenib dose. In patients with (n = 156) or without (n = 58) dose reduction, there was no difference in survival time (14(9.3–18) vs. 14 (8.9–20) months; p = 0.54).

There was no association between pre-sarcopenia and sorafenib treatment duration or dose reduction. Treatment duration of patients with pre-sarcopenia (n = 123) did not differ from the duration of patients without pre-sarcopenia (n = 91) (4.2(2.9–4.8) vs. 3.2(2.7–4.3); p = 0.76), and there was no difference in dose reduction frequency of pre-sarcopenia (75%) compared to patients without pre-sarcopenia (71%) (p = 0.64).

### Prognostic factors

The prognostic factors associated with OS were assessed in univariate and multivariate analyses. Univariate analysis revealed the following variables as significant prognostic factors: a baseline serum albumin level of ≤3.5 g/dl, a baseline AFP level of ≥100 ng/ml, a baseline AFP L3 fraction of ≥10%, a baseline PIVKA-II level of ≥100 mAU/ml, size of intrahepatic lesion ≥60 mm, presence of lesions in bilateral hepatic lobes, and presence of major portal vein invasion. We analyzed prognosis with the Barcelona Clinic Liver Cancer (BCLC) stage and ECOG performance status, but these factors were not prognostic factors: BCLC stage B (n = 109) vs. stage C (n = 105) (p = 0.09), and ECOG performance status 0(n = 116) vs. 1(n = 108) (p = 0.46). Multivariate analysis revealed a baseline serum albumin level of ≤3.5 g/dl [hazard ratio (HR) 1.9], a baseline AFP level of ≥100 ng/ml [HR 2.1], presence of lesions in bilateral hepatic lobes [HR 1.7], and presence of major portal vein invasion [HR 1.8] to be independent prognostic factors (Table 3). The albumin cut-off value of 3.5 g/dl was used based on the cut-off value in Child-Pugh classification. AFP level of ≥100 ng/ml is a standard cut-off value for the survival prediction of HCC patients, and we used this common cut-off value in this study.

**Table 3:** Multivariable analysis of prognostic factors in patients treated with sorafenib.

	Hazard Ratio	95% CI	*P* value
Albumin ≤3.5 (g/dl)	1.9	1.9–2.8	0.0006
AFP ≥100 (ng/ml)	2.1	1.3–3.4	0.002
Lesions in bilateral hepatic lobes	1.7	1.1–2.7	0.03
Major portal vein invasion	1.8	1.2–2.9	0.01

CI, confidence interval; AFP, alpha-fetoprotein; AFP L3 fraction.

**Fig 2:**
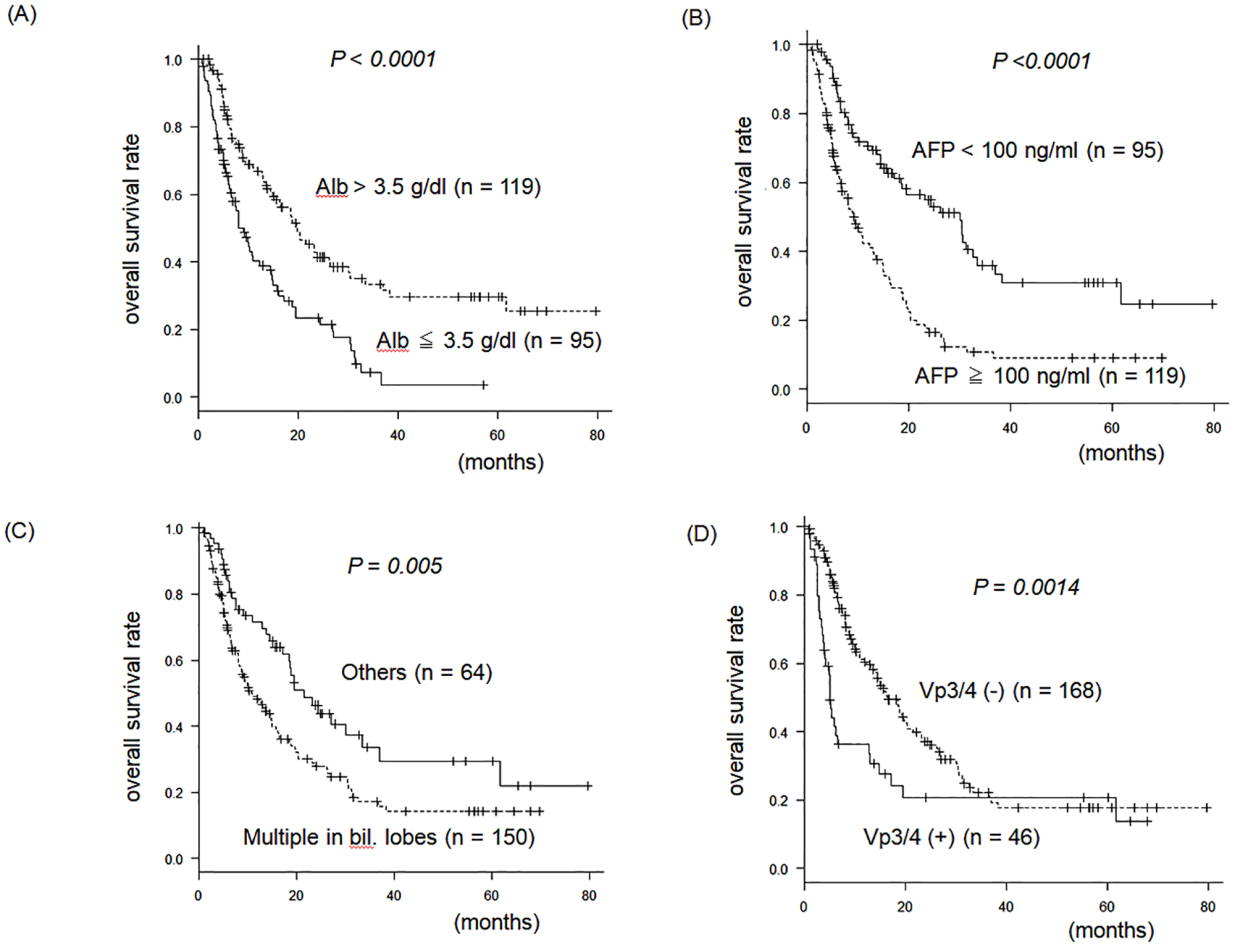
Prognostic factors in patients treated with sorafenib. AFP, alpha-fetoprotein; bil. lobes, bilateral lobes; Vp3/4, Major portal vein invasion.

**Fig 3:**
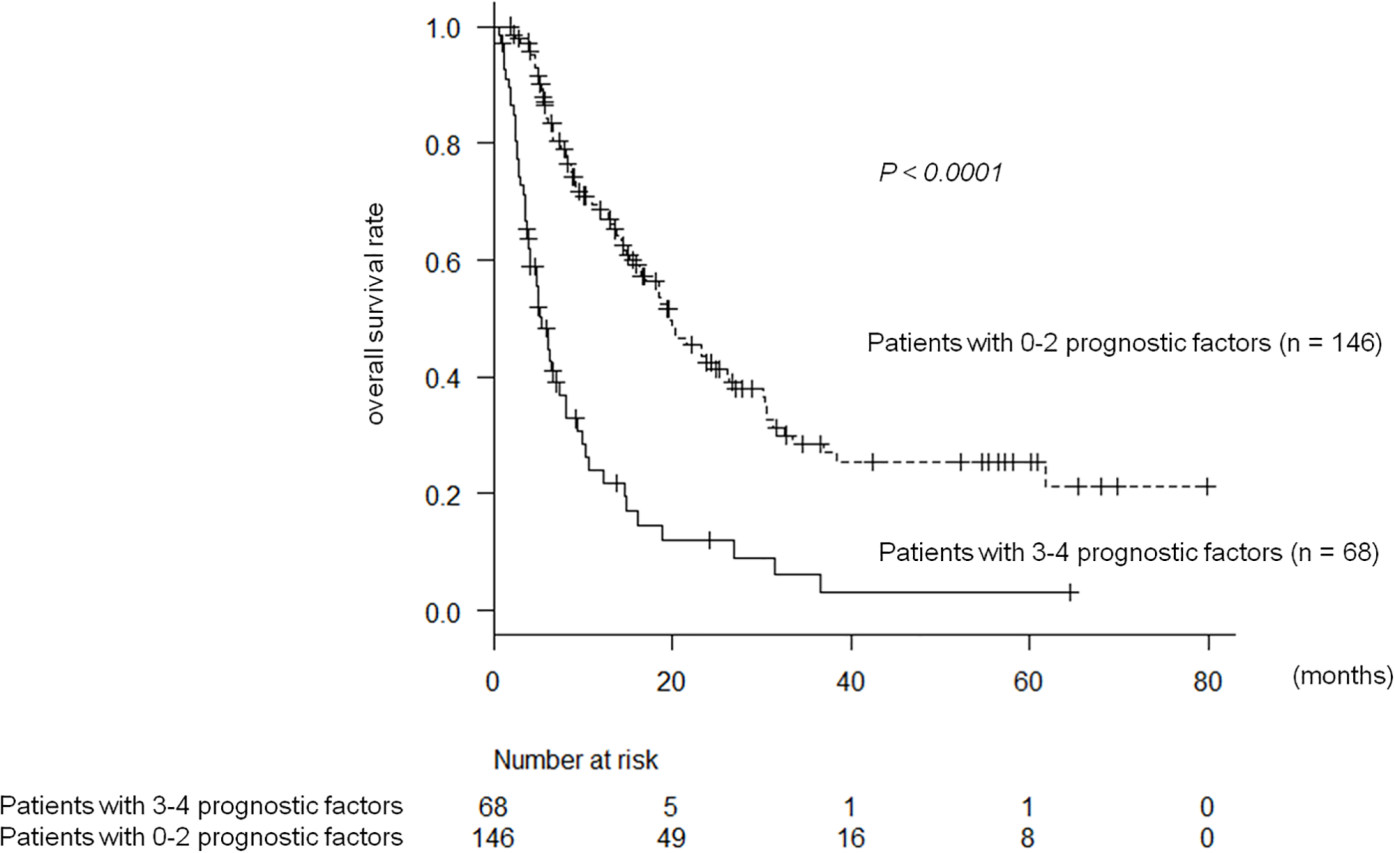
Overall survival in patients treated with sorafenib; patients with 0–2 prognostic factors versus patients with 3–4 prognostic factors.

MST stratified by each of these four prognostic factors were as follows: 18.8(14.4–24) vs. 8.1(6.3–11) months for Alb >3.5 vs. ≤3.5 g/dl; 24(16–33) vs. 9.1(6.3–12.2) months for AFP <100 vs. ≥100 ng/ml; 21(17–33) vs. 10(8.1–14) months for the absence vs. presence of lesions in bilateral hepatic lobes; and 16(13–20) vs. 5.2(3.8–6.5) months for the absence vs. presence of major portal vein invasion, respectively (Fig 2). The numbers of negative prognostic factors in each case were 0, 1, 2, 3, and 4 in 23, 54, 69, 54, and 14 patients, respectively. In patients having three or four prognostic factors, OS was significantly worse than those having two or less prognostic factors: 5.2(4.1–6.9) vs. 20(15–24) months [HR 3.3; 95% confidence interval (CI), 2.4–4.8; *p* < 0.0001] (Fig 3). The cut-off value for number of negative prognostic factors was calculated from the area under the curve analysis of progression-free survival and the number of negative factors.

### Impact of pre-sarcopenia on overall survival after stratification by prognostic factors

**Fig 4:**
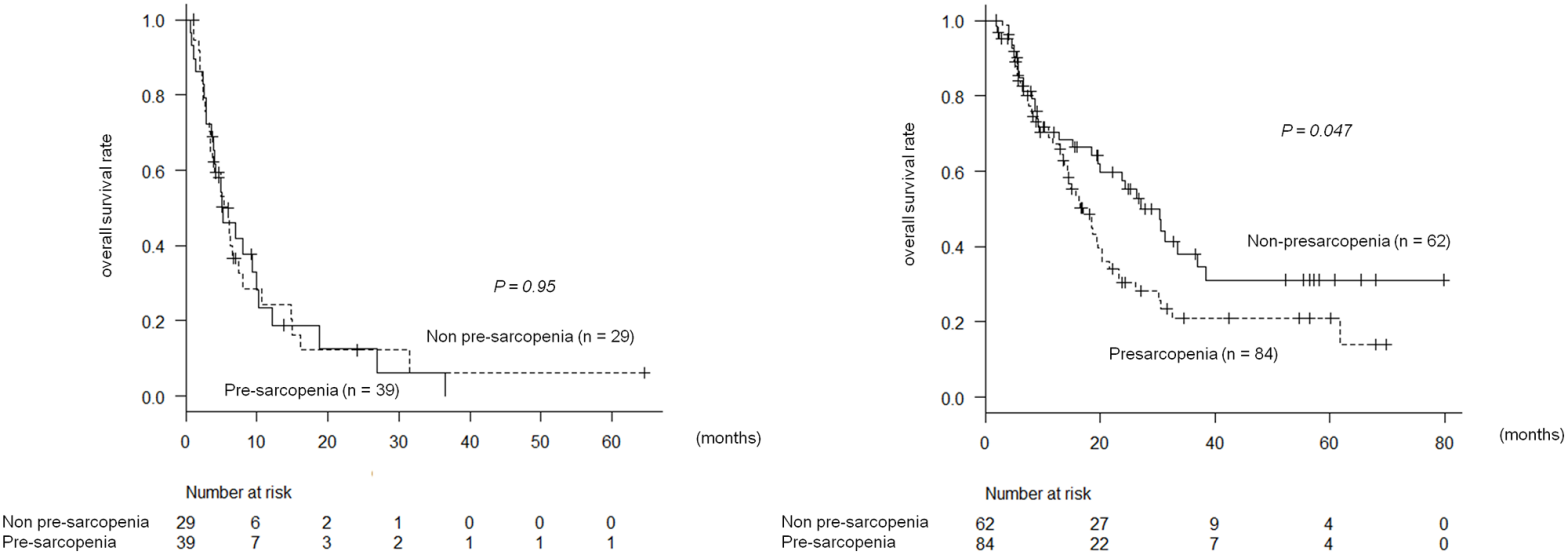
A. Presarcopenia and overall survival in patients with 3 or more prognostic factors. B. Presarcopenia and overall survival in patients with 0–2 prognostic factors.

In the 68 patients who had three or more negative prognostic factors, the presence of pre-sarcopenia did not correlate with prognosis (Fig 4A). However, of the 146 patients who had two or less prognostic factors, OS was significantly worse in 84 patients (58%) with pre-sarcopenia than in 62 patients without pre-sarcopenia. MST was 417 vs. 562 days and 6-, 12-, and 18-month survival rates were 79%, 58%, and 36% vs. 82%, 60%, and 52%, respectively (*p* = 0.047) (Fig 4B). According to Cox proportional hazard analysis, HR of pre-sarcopenia was 1.6 (95% CI, 1.0–2.4; *p* = 0.047).

## Discussion

In the present study, we evaluated the prevalence and significance of pre-sarcopenia in patients with advanced stage HCC treated with sorafenib, because sarcopenia or pre-sarcopenia has attracted attention as factors predicting poor prognosis in patients with chronic liver disease and HCC. The incidence of pre-sarcopenia in our study cohort was 57%, which is high compared with the reported prevalence of 15% in patients with chronic hepatitis and 24% in patients with compensated cirrhosis [2]. OS in patients with pre-sarcopenia tended to be slightly worse than in patients without pre-sarcopenia. Furthermore, when patients were stratified according to the number of traditional prognostic factors, the presence of pre-sarcopenia was significantly associated with poor prognosis in patients having no or few negative prognostic factors. This is the first report to define a subgroup of patients where pre-sarcopenia impacts prognosis.

There are several reports on the correlation between pre-sarcopenia and prognosis in patients with HCC who underwent resection [10, 33–35] or liver transplantation [12, 36, 37]. However, the evidence in patients with advanced HCC treated with sorafenib remains scarce. Previously, Hiraoka *et al*. reported that pre-sarcopenia was a negative prognostic factor independent of tumor factors and hepatic reserve in patients who received sorafenib treatment [15]. Similar to our present study, the authors examined the correlation between pre-sarcopenia, determined using CT and psoas muscle area index (PSI), and survival in 93 patients who received sorafenib treatment. The cut off value of PSI used was 4.24 cm^2^/m^2^ for men and 2.50 cm^2^/m^2^ for women, which was previously shown to be a prognostic factor in patients with chronic hepatic diseases in that institution. Pre-sarcopenia was found in 21.5% of patients and was an independent prognostic factor in addition to PIVKA-II level of ≥100 mAU/ml. In our present study, the incidence of pre-sarcopenia was as high as 57%, possibly because the study subjects included a larger number of elderly patients or those with decreased hepatic function than the previous study.

The difference between our present study and the previous study [15] was that survival, as determined by the presence of pre-sarcopenia, did not reach a statistically significant difference when analyzed in the whole cohort in our study, whereas pre-sarcopenia was a significant factor in the previous study. A possible reason for this difference may be differences in HCC conditions. Specifically, the incidence of extrahepatic metastasis and major vessel invasion was similar but the size and number of intrahepatic lesions were larger, and there were more advanced cases based on the TNM stage classification in our present study. Accordingly, we further investigated the significance of pre-sarcopenia by dividing the patients into two groups according to four negative prognostic factors, including baseline serum albumin levels, AFP levels, presence of lesions in bilateral hepatic lobes, and presence of major portal vein invasion. In patients having three or more negative prognostic factors, there was no significant correlation between pre-sarcopenia and prognosis. In contrast, in patients with better conditions having two or less prognostic factors, the prognosis was significantly worse in patients with pre-sarcopenia than in patients without pre-sarcopenia. Our present study suggested that although hepatic reserve and tumor conditions are the most important factors for predicting prognosis in patients undergoing sorafenib treatment for advanced HCC, pre-sarcopenia is also an important prognostic factor. In patients with 3 or more negative factors, the survival curve declined rapidly within the initial several months and MST was only 5.2 months. On the other hand, in patients with 2 or less negative factors, the decline in the survival curve was mild within the initial several month, which became further gradual after 10 weeks in patients without pre-sarcopenia. Therefore, the early decline in survival is primarily dependent on the number of negative factors whereas survival on the long term may be affected by the presence or absence of pre-sarcopenia. From these observations, we believe that pre-sarcopenia do not impact the survival in patients with 3 or more negative factors whose survival is very short. These findings suggest that evaluation of muscle volume could be one of the predictors and that intervention to improve pre-sarcopenia, such as nutritional therapy or exercise may help to improve prognosis, which need to be evaluated in the future studies.

Molecular mechanisms for the development of sarcopenia and its association with sorafenib treatment are largely unknown. Previous reports have suggested an involvement of reduced activation of the phosphatidylinositol 3-kinase-Akt-mammalian target of rapamycin (mTOR) pathway, which promotes protein synthesis, in the development of sarcopenia. For this pathway, it is reported that sorafenib treatment decreases protein synthesis by directly inhibiting the mTOR pathway by inhibiting vascular endothelial growth factor receptor. It may be possible that in patients who receive sorafenib treatment, these pathways lead to the development of sarcopenia leading to progression of cachexia, thereby worsening prognosis [21]. For the treatment of sarcopenia, nutrition, exercise, and medication including BCAA supplementation are recommended [38–41]. Therefore, these interventions may improve the prognosis in patients with pre-sarcopenia treated with sorafenib. It may be necessary to prospectively evaluate this in the future.

In some reports, it has been suggested that fat inside the muscle instead of muscle volume and decreased grip strength or physical function may contribute to the development of sarcopenia [7, 42]. Based on limitations of our present study, including that this was a retrospective study, measurement of grip strength or walking speed was not performed, and a relationship between sarcopenia and prognosis could not be analyzed. The fat inside the muscle was not evaluated. Further studies are required in the future. Another limitation is that the muscle volume measured by sliceOmatic and by different software programs were not compared because other software programs were not available.

In conclusion, in sorafenib treatment for advanced HCC, pre-sarcopenia is a prognostic factor in patients having no or few negative prognostic factors. Therefore, pre-sarcopenia could be the target of intervention to improve prognosis.

## References

[1] Cruz-Jentoft AJ, Landi F, Schneider SM, Zuniga C, Arai H, Boirie Y, et al Prevalence of and interventions for sarcopenia in ageing adults: a systematic review. Report of the International Sarcopenia Initiative (EWGSOP and IWGS). Age Ageing. 2014;43(6):748–59. doi: 10.1093/ageing/afu115 . 25241753 10.1093/ageing/afu115 PMC4204661

[2] Hiraoka A, Aibiki T, Okudaira T, Toshimori A, Kawamura T, Nakahara H, et al Muscle atrophy as pre-sarcopenia in Japanese patients with chronic liver disease: computed tomography is useful for evaluation. J Gastroenterol. 2015;50(12):1206–13. doi: 10.1007/s00535-015-1068-x . 25820219 10.1007/s00535-015-1068-x PMC4673094

[3] Kalafateli M, Konstantakis C, Thomopoulos K, Triantos C. Impact of muscle wasting on survival in patients with liver cirrhosis. World J Gastroenterol. 2015;21(24):7357–61. doi: 10.3748/wjg.v21.i24.7357 . 26139982 10.3748/wjg.v21.i24.7357 PMC4481431

[4] Iritani S, Imai K, Takai K, Hanai T, Ideta T, Miyazaki T, et al Skeletal muscle depletion is an independent prognostic factor for hepatocellular carcinoma. J Gastroenterol. 2015;50(3):323–32. doi: 10.1007/s00535-014-0964-9 . 24817668 10.1007/s00535-014-0964-9

[5] Montano-Loza AJ, Duarte-Rojo A, Meza-Junco J, Baracos VE, Sawyer MB, Pang JX, et al Inclusion of Sarcopenia Within MELD (MELD-Sarcopenia) and the Prediction of Mortality in Patients With Cirrhosis. Clin Transl Gastroenterol. 2015;6(6):e102 doi: 10.1038/ctg.2015.31 . 26181291 10.1038/ctg.2015.31 PMC4816259

[6] Montano-Loza AJ. Clinical relevance of sarcopenia in patients with cirrhosis. World J Gastroenterol. 2014;20(25):8061–71. doi: 10.3748/wjg.v20.i25.8061 . 25009378 10.3748/wjg.v20.i25.8061 PMC4081677

[7] Montano-Loza AJ, Angulo P, Meza-Junco J, Prado CM, Sawyer MB, Beaumont C, et al Sarcopenic obesity and myosteatosis are associated with higher mortality in patients with cirrhosis. J Cachexia Sarcopenia Muscle. 2016;7(2):126–35. doi: 10.1002/jcsm.12039 . 27493866 10.1002/jcsm.12039 PMC4864157

[8] Hara N, Iwasa M, Sugimoto R, Mifuji-Moroka R, Yoshikawa K, Terasaka E, et al Sarcopenia and Sarcopenic Obesity Are Prognostic Factors for Overall Survival in Patients with Cirrhosis. Intern Med. 2016;55(8):863–70. doi: 10.2169/internalmedicine.55.5676 . 27086797 10.2169/internalmedicine.55.5676

[9] Nishikawa H, Osaki Y. Liver Cirrhosis: Evaluation, Nutritional Status, and Prognosis. Mediators Inflamm. 2015;2015(10):872152 doi: 10.1155/2015/872152 . 26494949 10.1155/2015/872152 PMC4606163

[10] Harimoto N, Yoshizumi T, Shimokawa M, Sakata K, Kimura K, Itoh S, et al Sarcopenia is a poor prognostic factor following hepatic resection in patients aged 70 years and older with hepatocellular carcinoma. Hepatol Res. 2016;46(12):1247–55. doi: 10.1111/hepr.12674 . 26880049 10.1111/hepr.12674

[11] Valero V 3rd, Amini N, Spolverato G, Weiss MJ, Hirose K, Dagher NN, et al Sarcopenia adversely impacts postoperative complications following resection or transplantation in patients with primary liver tumors. J Gastrointest Surg. 2015;19(2):272–81. doi: 10.1007/s11605-014-2680-4 . 25389056 10.1007/s11605-014-2680-4 PMC4332815

[12] Kaido T, Tamai Y, Hamaguchi Y, Okumura S, Kobayashi A, Shirai H, et al Effects of pretransplant sarcopenia and sequential changes in sarcopenic parameters after living donor liver transplantation. Nutrition. 2017;33:195–8. doi: 10.1016/j.nut.2016.07.002 . 27649861 10.1016/j.nut.2016.07.002

[13] Dodson RM, Firoozmand A, Hyder O, Tacher V, Cosgrove DP, Bhagat N, et al Impact of sarcopenia on outcomes following intra-arterial therapy of hepatic malignancies. J Gastrointest Surg. 2013;17(12):2123–32. doi: 10.1007/s11605-013-2348-5 . 24065364 10.1007/s11605-013-2348-5 PMC3982291

[14] Llovet JM, Ricci S, Mazzaferro V, Hilgard P, Gane E, Blanc JF, et al Sorafenib in advanced hepatocellular carcinoma. N Engl J Med. 2008;359(4):378–90. doi: 10.1056/NEJMoa0708857 . 18650514 10.1056/NEJMoa0708857

[15] Hiraoka A, Hirooka M, Koizumi Y, Izumoto H, Ueki H, Kaneto M, et al Muscle volume loss as a prognostic marker in hepatocellular carcinoma patients treated with sorafenib. Hepatol Res. 2016;1(10):12780 doi: 10.1111/hepr.12780 . 27480045 10.1111/hepr.12780

[16] Imai K, Takai K, Hanai T, Ideta T, Miyazaki T, Kochi T, et al Skeletal muscle depletion predicts the prognosis of patients with hepatocellular carcinoma treated with sorafenib. Int J Mol Sci. 2015;16(5):9612–24. doi: 10.3390/ijms16059612 . 25927582 10.3390/ijms16059612 PMC4463608

[17] Mir O, Coriat R, Blanchet B, Durand JP, Boudou-Rouquette P, Michels J, et al Sarcopenia predicts early dose-limiting toxicities and pharmacokinetics of sorafenib in patients with hepatocellular carcinoma. PLoS One. 2012;7(5):e37563 doi: 10.1371/journal.pone.0037563 . 22666367 10.1371/journal.pone.0037563 PMC3364283

[18] Bruix J, Sherman M. Management of hepatocellular carcinoma: an update. Hepatology. 2011;53(3):1020–2. doi: 10.1002/hep.24199 . 21374666 10.1002/hep.24199 PMC3084991

[19] Llovet JM, Bru C, Bruix J. Prognosis of hepatocellular carcinoma: the BCLC staging classification. Semin Liver Dis. 1999;19(3):329–38. doi: 10.1055/s-2007-1007122 . 10518312 10.1055/s-2007-1007122

[20] Oken MM, Creech RH, Tormey DC, Horton J, Davis TE, McFadden ET, et al Toxicity and response criteria of the Eastern Cooperative Oncology Group. Am J Clin Oncol. 1982;5(6):649–55. . 7165009

[21] Antoun S, Lanoy E, Iacovelli R, Albiges-Sauvin L, Loriot Y, Merad-Taoufik M, et al Skeletal muscle density predicts prognosis in patients with metastatic renal cell carcinoma treated with targeted therapies. Cancer. 2013;119(18):3377–84. doi: 10.1002/cncr.28218 . 23801109 10.1002/cncr.28218

[22] Morimoto M, Numata K, Kondo M, Hidaka H, Takada J, Shibuya A, et al Higher discontinuation and lower survival rates are likely in elderly Japanese patients with advanced hepatocellular carcinoma receiving sorafenib. Hepatol Res. 2011;41(4):296–302. doi: 10.1111/j.1872-034X.2011.00778.x . 21348907 10.1111/j.1872-034X.2011.00778.x

[23] Kim JE, Ryoo BY, Ryu MH, Chang HM, Suh DJ, Lee HC, et al Sorafenib dose escalation in the treatment of advanced hepatocellular carcinoma. Oncology. 2012;82(2):119–25. doi: 10.1159/000336082 . 22354124 10.1159/000336082

[24] Nishikawa H, Osaki Y, Endo M, Takeda H, Tsuchiya K, Joko K, et al Comparison of standard-dose and halfdose sorafenib therapy on clinical outcome in patients with unresectable hepatocellular carcinoma in field practice: A propensity score matching analysis. Int J Oncol. 2014;45(6):2295–302. doi: 10.3892/ijo.2014.2654 . 25230744 10.3892/ijo.2014.2654

[25] Antoun S, Baracos VE, Birdsell L, Escudier B, Sawyer MB. Low body mass index and sarcopenia associated with dose-limiting toxicity of sorafenib in patients with renal cell carcinoma. Ann Oncol. 2010;21(8):1594–8. doi: 10.1093/annonc/mdp605 . 20089558 10.1093/annonc/mdp605

[26] Eisenhauer EA, Therasse P, Bogaerts J, Schwartz LH, Sargent D, Ford R, et al New response evaluation criteria in solid tumours: revised RECIST guideline (version 1.1). Eur J Cancer. 2009;45(2):228–47. doi: 10.1016/j.ejca.2008.10.026 . 19097774 10.1016/j.ejca.2008.10.026

[27] Lencioni R, Llovet JM. Modified RECIST (mRECIST) assessment for hepatocellular carcinoma. Semin Liver Dis. 2010;30(1):52–60. doi: 10.1055/s-0030-1247132 . 20175033 10.1055/s-0030-1247132 PMC12268942

[28] Prado CM, Birdsell LA, Baracos VE. The emerging role of computerized tomography in assessing cancer cachexia. Curr Opin Support Palliat Care. 2009;3(4):269–75. doi: 10.1097/SPC.0b013e328331124a . 19667996 10.1097/SPC.0b013e328331124a

[29] Nishikawa H, Shiraki M, Hiramatsu A, Moriya K, Hino K, Nishiguchi S. Japan Society of Hepatology guidelines for sarcopenia in liver disease (1st edition): Recommendation from the working group for creation of sarcopenia assessment criteria. Hepatol Res. 2016;46(10):951–63. doi: 10.1111/hepr.12774 . 27481650 10.1111/hepr.12774

[30] Sato Y, Nakata K, Kato Y, Shima M, Ishii N, Koji T, et al Early recognition of hepatocellular carcinoma based on altered profiles of alpha-fetoprotein. N Engl J Med. 1993;328(25):1802–6. doi: 10.1056/NEJM199306243282502 . 7684823 10.1056/NEJM199306243282502

[31] Kumada T, Toyoda H, Kiriyama S, Tanikawa M, Hisanaga Y, Kanamori A, et al Predictive value of tumor markers for hepatocarcinogenesis in patients with hepatitis C virus. J Gastroenterol. 2011;46(4):536–44. doi: 10.1007/s00535-010-0349-7 . 21132575 10.1007/s00535-010-0349-7

[32] Kanda Y. Investigation of the freely available easy-to-use software 'EZR' for medical statistics. Bone Marrow Transplant. 2013;48(3):452–8. doi: 10.1038/bmt.2012.244 . 23208313 10.1038/bmt.2012.244 PMC3590441

[33] Kobayashi A, Kaido T, Hamaguchi Y, Okumura S, Taura K, Hatano E, et al Impact of postoperative changes in sarcopenic factors on outcomes after hepatectomy for hepatocellular carcinoma. J Hepatobiliary Pancreat Sci. 2016;23(1):57–64. doi: 10.1002/jhbp.302 . 26572789 10.1002/jhbp.302

[34] Itoh S, Shirabe K, Matsumoto Y, Yoshiya S, Muto J, Harimoto N, et al Effect of body composition on outcomes after hepatic resection for hepatocellular carcinoma. Ann Surg Oncol. 2014;21(9):3063–8. doi: 10.1245/s10434-014-3686-6 . 24719020 10.1245/s10434-014-3686-6

[35] Fujiwara N, Nakagawa H, Kudo Y, Tateishi R, Taguri M, Watadani T, et al Sarcopenia, intramuscular fat deposition, and visceral adiposity independently predict the outcomes of hepatocellular carcinoma. J Hepatol. 2015;63(1):131–40. doi: 10.1016/j.jhep.2015.02.031 . 25724366 10.1016/j.jhep.2015.02.031

[36] Tandon P, Ney M, Irwin I, Ma MM, Gramlich L, Bain VG, et al Severe muscle depletion in patients on the liver transplant wait list: its prevalence and independent prognostic value. Liver Transpl. 2012;18(10):1209–16. doi: 10.1002/lt.23495 . 22740290 10.1002/lt.23495

[37] Kaido T. Selection Criteria and Current Issues in Liver Transplantation for Hepatocellular Carcinoma. Liver Cancer. 2016;5(2):121–7. doi: 10.1159/000367749 . 27386430 10.1159/000367749 PMC4906426

[38] Roman E, Torrades MT, Nadal MJ, Cardenas G, Nieto JC, Vidal S, et al Randomized pilot study: effects of an exercise programme and leucine supplementation in patients with cirrhosis. Dig Dis Sci. 2014;59(8):1966–75. doi: 10.1007/s10620-014-3086-6 . 24599772 10.1007/s10620-014-3086-6

[39] Thandassery RB, Montano-Loza AJ. Role of Nutrition and Muscle in Cirrhosis. Curr Treat Options Gastroenterol. 2016;14(2):257–73. doi: 10.1007/s11938-016-0093-z . 27023701 10.1007/s11938-016-0093-z

[40] Hayashi F, Matsumoto Y, Momoki C, Yuikawa M, Okada G, Hamakawa E, et al Physical inactivity and insufficient dietary intake are associated with the frequency of sarcopenia in patients with compensated viral liver cirrhosis. Hepatol Res. 2013;43(12):1264–75. doi: 10.1111/hepr.12085 . 23489325 10.1111/hepr.12085

[41] Kappus MR, Mendoza MS, Nguyen D, Medici V, McClave SA. Sarcopenia in Patients with Chronic Liver Disease: Can It Be Altered by Diet and Exercise? Curr Gastroenterol Rep. 2016;18(8):43 doi: 10.1007/s11894-016-0516-y . 27372291 10.1007/s11894-016-0516-y

[42] Hamaguchi Y, Kaido T, Okumura S, Kobayashi A, Fujimoto Y, Ogawa K, et al Muscle Steatosis is an Independent Predictor of Postoperative Complications in Patients with Hepatocellular Carcinoma. World J Surg. 2016;40(8):1959–68. doi: 10.1007/s00268-016-3504-3 . 27071610 10.1007/s00268-016-3504-3

